# Classic Bioelectrical Impedance Vector Reference Values for Assessing Body Composition in Male and Female Athletes

**DOI:** 10.3390/ijerph16245066

**Published:** 2019-12-12

**Authors:** Francesco Campa, Catarina Matias, Hannes Gatterer, Stefania Toselli, Josely C. Koury, Angela Andreoli, Giovanni Melchiorri, Luis B. Sardinha, Analiza M. Silva

**Affiliations:** 1 Departments of Biomedical and Neuromotor Sciences, University of Bologna, 40121 Bologna, Italy; francesco.campa3@unibo.it; 2 Exercise and Health Laboratory, CIPER, Faculdade de Motricidade Humana, Universidade de Lisboa, 1499-002 Cruz Quebrada, Portugal; cmatias@fmh.ulisboa.pt (C.M.); lbsardinha55@gmail.com (L.B.S.); analiza.monica@gmail.com (A.M.S.); 3 Institute of Mountain Emergency Medicine, Eurac Research, 40121 Bolzano, Italy; Hannes.Gatterer@eurac.edu; 4 Department of Basic and Experimental Nutrition, Nutrition Institute, State University of Rio de Janeiro, Rio de Janeiro 20550-900, Brazil; jckoury@gmail.com; 5 Department of Systems Medicine, University of Tor Vergata, 00175 Rome, Italy; angela.andreoli@uniroma2.it (A.A.); gmelchiorri@libero.it (G.M.)

**Keywords:** BIVA, confidence ellipses, phase angle, R–Xc graph, tolerance ellipses

## Abstract

Bioimpedance standards are well established for the normal healthy population and in clinical settings, but they are not available for many sports categories. The aim of this study was to develop reference values for male and female athletes using classic bioimpedance vector analysis (BIVA). In this study, 1556 athletes engaged in different sports were evaluated during their off-season period. A tetrapolar bioelectrical impedance analyzer was used to determine measurements of resistance (R) and reactance (Xc). The classic BIVA procedure, which corrects bioelectrical values for body height, was applied, and fat-free mass, fat mass, and total body water were estimated. In order to verify the need for specific references, classic bioelectrical values were compared to the reference values for the general male and female populations. Additionally, athletes were divided into three groups: endurance, velocity/power, and team sports. In comparison with the general healthy male and female populations, the mean vectors of the athletes showed a shift to the left on the R–Xc graph. Considering the same set of modalities, BIVA confidence graphs showed that male and female endurance athletes presented lower body fluids, fat mass, and fat-free mass than other sets of modalities. This study provides BIVA reference values for an athletic population that can be used as a standard for assessing body composition in male and female athletes.

## 1. Introduction

The analysis of body composition (BC) is a critical component in the sports field, given its relationship to physical performance [1]. Several techniques are used to assess BC in athletes. These include underwater weighing (densitometry), dual energy X-ray absorptiometry (DXA), bioelectrical impedance analysis (BIA), and anthropometric measurements. Although densitometry and DXA are the most accurate methods to evaluate BC, they are expensive and impractical for field use because they require large, specialized equipment. In recent years, bioimpedance vector analysis (BIVA) has received attention in the sports science field as a method to obtain a qualitative assessment of BC [2].

BIA data (i.e., resistance (R) and reactance (Xc)), through BIVA, are used to evaluate cellular function and body fluid content. This method plots the impedance parameters standardized for the subject’s height as a single vector on the R–Xc graph [3,4]. Recently, different studies compared BIVA, dual X-ray absorptiometry, and body fluid measurements obtained by dilution technique. The studies demonstrated the ability of BIVA to evaluate BC changes in athletes by studying the vector displacements and BC variables (i.e., total body water (TBW), percentage of fat mass (%FM), and the intracellular/extracellular water (ICW/ECW) ratio) [5,6]. The ICW/ECW ratio is identified by the bioelectrical phase angle (PA), which is calculated as the arctangent of Xc/R × 180°/π [5,6,7]. An increase in ICW/ECW can be a consequence of gain in muscle mass due to physical activity [8,9,10,11]. However, the analysis of only the PA leads to interpretation errors. In fact, groups of athletes characterized by a similar PA may show different TBW or %FM. BIVA goes beyond this limitation because it takes into consideration the relationship between ICW and ECW (determined by the vector slope in the R–Xc graph) in addition to the TBW amount (which is represented by the vector length) [5].

Recent studies conducted on athletes have highlighted the need for sport-specific BIVA references by establishing data from sets of sports such as soccer, volleyball, or cycling [12,13,14]. In the absence of appropriate references for each sport category, researchers continue to use reference values from the general healthy population [15,16,17,18]. A characteristic and innovative aspect of BIVA is that it provides soft tissue classification (under, normal, and over) and ranking (more or less than before intervention), comparing the position of an individual vector to a reference population. For this reason, the application of BIVA can be compromised if inappropriate reference R–Xc graphs are used, leading to evaluations that are difficult to interpret. Furthermore, to the best of our knowledge, no reference values are available for female athletes.

The use of appropriate R–Xc graphs allows for the correct interpretation of BIVA patterns in the assessment of BC, adding useful information regarding the influence of BC on somatic maturation in young athletes [19]. While specific references do not exist for every single sport, establishing BIVA references for the general male and female athlete populations may be useful even if values may differ slightly between individual sports. Considering the importance of the use of BIVA for the athletic population, the present study aimed to generate bioimpedance reference values for male and female athletes.

## 2. Materials and Methods

### 2.1. Subjects

This was a cross-sectional observational study on 1556 athletes (men: n = 1116, age 23.1 ± 6.8 y; women: n = 440, age 26.9 ± 6.6 y). The athletes participated in 23 sport modalities: athletics (men = 78 and women = 19), badminton (men = 5 and women = 4), basketball (men = 117 and women = 53), boxing (women = 9), cross-country skiing (men = 4), CrossFit (men = 26 and women = 33), cycling (men = 15), field hockey (men = 12), handball (men = 43 and women = 4), judo (men = 78 and women = 28), karate (men = 29 and women = 5), kick boxing (men = 48 and women = 20), marathon (men = 49 and women = 24), pentathlon (men = 33 and women = 21), rowing (women = 13), rugby (men = 102), rhythmic gymnastics (women = 28), soccer (men = 67), short-distance swimming (men = 85 and women = 49), tennis (men = 26 and women = 15), triathlon (men = 64 and women = 18), volleyball (men = 176 and women = 79), and water polo (men = 59 and women = 17).

The control groups were represented by the healthy male and female general populations [20]. The athletes were sorted into three groups: endurance (cycling, marathon, pentathlon, cross-country skiing, rowing, and triathlon), velocity/power (athletics including jumping, throwing, short-distance running; badminton; boxing; CrossFit; judo; karate; kickboxing; rhythmic gymnastics; swimming including short-distance swimming; and tennis), and team sports (basketball, field hockey, handball, rugby, soccer, volleyball, and water polo).

Medical screening indicated that all subjects were in good health. The following inclusion criteria were used: (1) a minimum of 10 to a maximum of 13 h of training per week; (2) tested negative for performance-enhancing drugs; and (3) not taking any medications. All subjects were informed of the study procedures before they gave written consent to participate. All procedures were approved by the Bioethics Committee of the University of Bologna and were conducted in accordance with the Declaration of Helsinki for human studies (Ethical Approval Code: 25027; dated 13.03.2017).

### 2.2. Procedures

**Figure 1:**
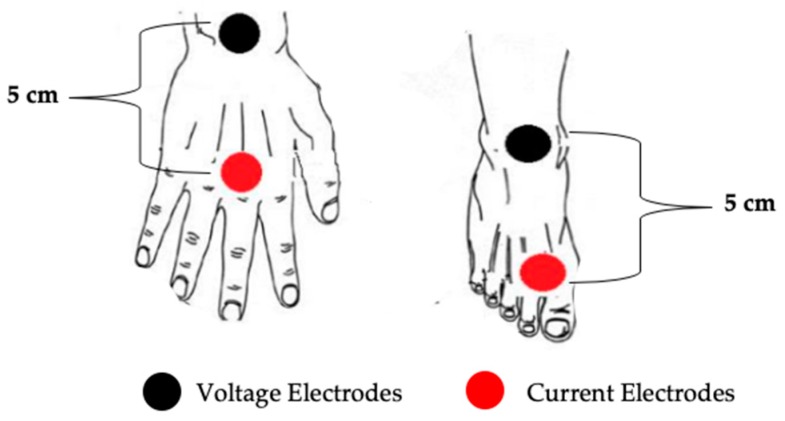
Hand and foot electrode positioning.

All measurements were performed in resting conditions during the off-season period (9:00 a.m.) at the athletes’ training centers. The impedance measurements were performed with BIA (BIA 101 Anniversary, Akern, Florence, Italy), which applies an alternating current of 800 μA at a single frequency of 50 kHz. Measurements were made using four electrical conductors. The subjects were in the supine position with a leg opening of 45° compared to the median line of the body and the upper limbs positioned 30° away from the trunk. After cleansing the skin with alcohol, two Ag/AgCl low-impedance electrodes (Biatrodes, Akern Srl, Florence, Italy) were placed on the back of the right hand and two electrodes on the corresponding foot, with a distance of 5 cm between each other [4,21] (Figure 1). To avoid disturbances in fluid distribution, athletes were instructed to abstain from food and drink for ≥4 hours before the test.

The bioelectrical PA was calculated as the arctangent of Xc/R × 180°/π. Total body water (TBW), fat-free mass (FFM) and fat mass (FM) were determined according to Kotler et al. [22]. The vector length (Z/H) was calculated as the hypotenuses of individual impedance normalized values. Height was measured to the nearest 0.1 cm using a stadiometer. Body weight was determined to the nearest 0.1 kg using a mechanical scale. Body mass index (BMI) was calculated as total body mass (kilograms) divided by height (meters) squared.

### 2.3. Statistical Analysis

Results are presented as mean ± SD. Normal distribution of data was evaluated using the Shapiro–Wilk test. A univariate analysis of variance (ANOVA) with Bonferroni post-hoc tests for multiple comparisons was performed. The two-sample Hotelling T^2^ test was used to compare the differences in the mean impedance vectors between groups. The 50%, 75%, and 95% tolerance ellipses were generated using the BIVA software [23]. Statistical significance was predetermined as *p* < 0.05. IBM SPSS 23.0 (SPSS, Chicago, IL, USA) was used for all statistical calculations.

## 3. Results

Characteristics of the athletes, sorted into three groups by sex and ANOVA results, are reported in Table 1. Male team-sports athletes showed higher values of height, weight, BMI, FM, FFM, and TBW than those for male endurance and male velocity/power athletes (*p* < 0.01). Female team-sports athletes showed higher values of weight, FFM, and TBW than female endurance and female velocity/power athletes (*p* < 0.01). FM was higher in female team-sports athletes than in female velocity/power athletes. For both sexes, bioelectrical variables were lower in team-sports athletes, except for PA, which was similar for all groups. 

**Figure 2:**
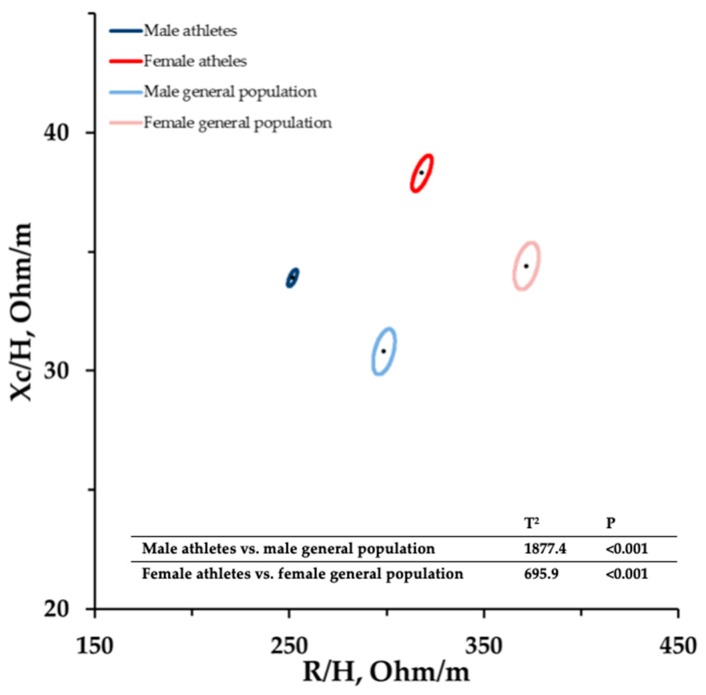
Mean impedance vectors with the 95% confidence ellipses for athletes and the general healthy populations. The Hotelling T^2^ test results are included.

Figure 2 shows the mean impedance vectors with the 95% confidence ellipses for all athletes compared to the 95% ellipses for the general healthy reference populations [20]. Compared to the general populations, the vectors of both male and female athletes shifted to the left.

**Figure 3:**
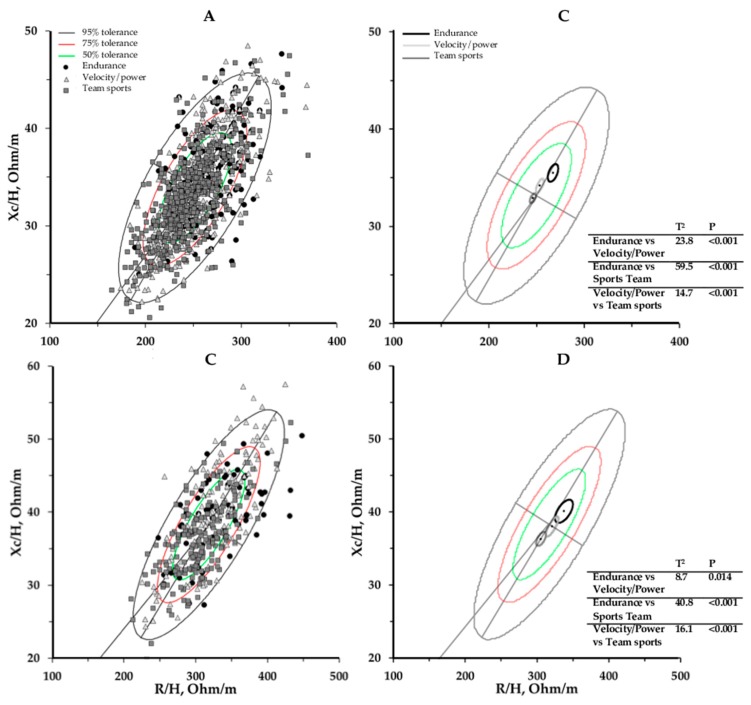
Scattergrams of the individual (**A**,**C**) and mean (**B**,**D**) impedance vectors, divided by sports categories and plotted on the new tolerance ellipses, for male (**A**,**B**) and female (**C**,**D**) athletes. The Hotelling T^2^ test results are included.

The mean impedance vectors of the three categories of athletes showed significant differences between each other (*p* < 0.01). Similarly, male and female endurance athletes showed longer impedance vectors than the athletes included in the velocity/power and team-sports groups, which were respectively lower in the R–Xc graph (Figure 3B,D).

**Figure 4:**
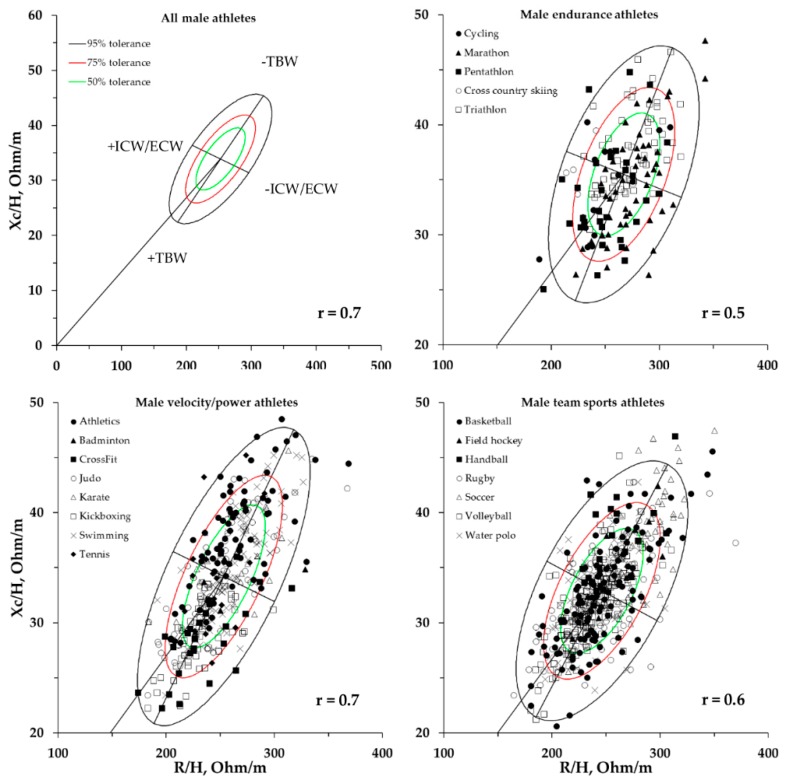
New 50%, 75%, and 95% tolerance ellipses of the entire male athlete population and for the endurance, velocity/power, and team-sports categories with the single athletes’ vectors. r = correlation coefficient between R/H and Xc/H. ICW/ECW: intracellular/extracellular water ratio.

**Figure 5:**
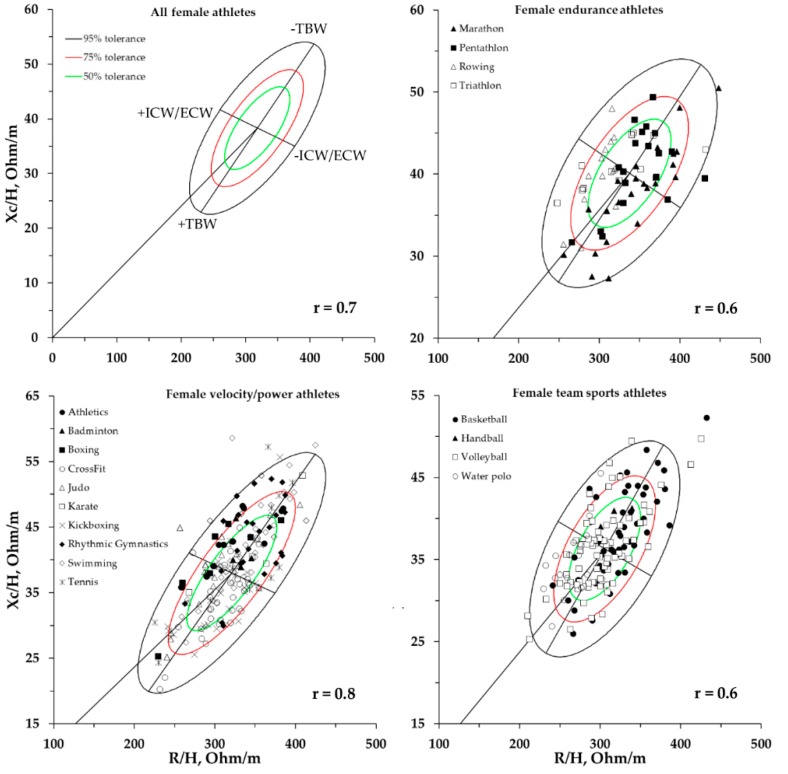
New 50%, 75%, and 95% tolerance ellipses of the entire female athlete population and for the endurance, velocity/power, and team-sports categories with the single athletes’ vectors. r = correlation coefficient between R/H and Xc/H.

The new 50%, 75%, and 95% tolerance ellipses calculated for all athletes and for the three groups are shown in Figure 4 for males and in Figure 5 for females.

**Figure 6:**
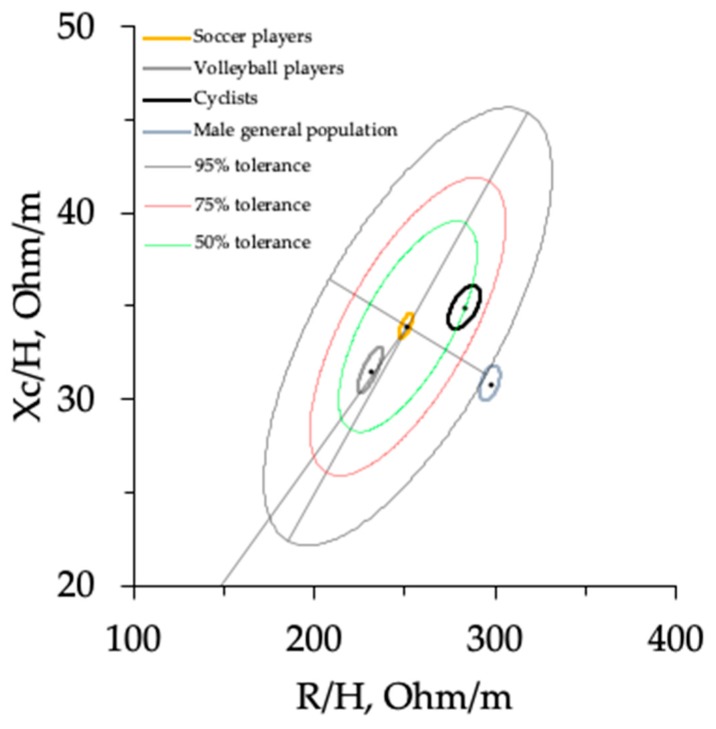
The 95% confidence ellipses for the mean impedance vectors of elite male soccer players [12], volleyball players [14], cyclists [15], and for the general population [19] plotted on the new reference tolerance ellipses.

The 95% confidence ellipses for the mean impedance vectors of elite male soccer players [12] (n = 219, R/H = 252.1, SD = 23.1, Xc/H = 33.9, SD = 4.1, r R/H; Xc/H = 0.69), volleyball players [14] (n = 75, R/H = 232.1, SD = 24.1, Xc/H = 31.5, SD = 4.3, r R/H; Xc/H = 0.81), and cyclists [13] (n = 79, R/H = 284.5, SD = 31.4, Xc/H = 34.9, SD = 4.1, r R/H; Xc/H = 0.56) plotted on the new reference tolerance ellipses are shown in Figure 6. Separate 95% confidence ellipses indicate a significant vector difference (*p* < 0.05).

## 4. Discussion

The present study, for the first time, generates BIVA reference data for the male and female athlete populations and for endurance, velocity/power, and team-sports athletes. The different distributions of the athletes’ bioelectrical values compared to the general population is indicative of the athletes’ BC peculiarities and shows the need for athlete-specific reference values. The differences in the BIVA distributions between athlete groups and the general healthy population might reflect the sport- and training-specific adaptation of body masses and composition. In fact, the athletes’ lower R/H and higher Xc/H values compared to the general population indicate a higher ICW/ECW ratio and FFM, which is typical for athletes [24,25].

Additionally, the comparison of the 95% confidence ellipses of the mean impedance vectors of the three groups (endurance, velocity/power, and team sports) shows that each athlete group had a slightly different vector distribution on the R–Xc graph. As previously noted, in addition to having morphological and BC differences compared to the general population [24,25], athletes also differ according to the sport that they practice [26,27,28]. In particular, compared to the velocity/power and team-sports athletes, endurance athletes showed a longer mean impedance vector on the R–Xc graph, indicating less body fluids, which is most likely due to lower body weight. On the contrary, athletes practicing in team sports are positioned at the lowest points on the R–Xc graph compared to the other two group categories.

Our results show that there were no existing PA differences among the three groups of athletes. Recently, a literature review conducted by Di Vincenzo et al. [29] suggested that the PA is a reliable indicator of the ICW/ECW ratio, and that it decreases with age and varies by sex [30]. However, the authors highlighted that it is still uncertain to what extent the PA varies between different sports. Our results show significant differences among the three groups of athletes assessed by BIVA, in both sexes. The BIVA vector position, whose slope is determined by the PA and whose length is determined by the TWB amount, improves the categorization of athletes practicing different sports by overcoming the limitation of evaluating only the PA. In fact, the reference data of the elite categories of soccer [12], volleyball [14], and cycling showed different vector displacements on the R–Xc graph when assessed by BIVAR–. In particular, if we consider the reference values of the PA in soccer players and volleyball players, they were closely similar (7.7 ± 0.6 and 7.7 ± 0.7, respectively). However, as shown in the R–Xc graph (Figure 6), the vector length was lower in volleyball players than in soccer players. This indicates an increased quantity of fluids due to a heavier body mass with similar ICW/ECW ratio. In this regard, as shown in a cross-sectional study by Campa et al. [31], high-level volleyball and soccer players present similar calf and thigh muscle areas, but upper muscle area and body mass are greater in athletes who play volleyball. As studied previously, BIVA has already shown effectiveness in discriminating between division levels within the same sport [12,13,14]. This is due to the fact that athletes competing at elite levels, in addition to better technical abilities, have a different BC with higher muscle masses and a lower %FM [12,31,32].

A strength of this study is the large sample size of 1116 males and 440 female athletes (in comparison to the 354 male and 372 female subjects proposed as reference for the general population by Piccoli et al. [20]). Additionally, subjects considered in this study were measured in the same conditions and during the same off-season period. This is noteworthy when comparing BIVA measurements because vector changes occur during the competitive season [6,15,33]. Future research should provide sport-specific BIVA references, including samples of different division categories, with a focus on the vector changes throughout the competitive period.

Some limitations of this study should also be acknowledged. Our results are only applicable when using bioimpedance devices operating at a single frequency of 50 kHz. Additionally, the proposed references are specific to the population that was tested in this study. Even though using these BIVA references might constitute an advantage to the use of the general population data, it still might not be the optimal solution for each individual sport.

## 5. Conclusions

This study proposes new BIVA references that will be useful in assessing BC in male and female athletes. The research shows that vector distributions of endurance, velocity/power, and team-sport athletes differ from the general healthy population and among themselves, due to their different BCs. The BIVA vector, whose position is due to the PA and vector length values, provides additional information with respect to the interpretation of the PA alone. This allows for the assessment of soft tissues (e.g., ICW/ECW) in relation to TBW.

## Figures and Tables

**Table 1:** Descriptive and comparative statistics for anthropometric, bioelectrical, and body composition variables for the athletes according to sex and sports categories.

Variable	Male					Female				
	Endurance n = 165	Velocity/Power n = 375	Team Sports n = 576	ANOVA *p*	All n = 1116	Endurance n = 76	Velocity/Power n = 177	Team Sports n = 187	ANOVA *p*	All n = 440
Age (years)	23.5 ± 6.2	23.6 ± 7.4	22.7 ± 6.5	0.098	23.1 ± 6.8	27.4 ± 7.0	26.2 ± 5.5	27.5 ± 7.4	0.116	26.9 ± 6.6
Weight (kg)	69.8 ± 8.1 ^#§^	74.7 ± 9.8 ^*§^	84.7 ± 13.8 *^#^	<0.001	79.5 ± 13.5	61.2 ± 8.4 ^§^	62.5 ± 7.9 ^§^	68.5 ± 9.1 *^#^	<0.001	65.4 ± 9.2
Height (cm)	175.9 ± 6.2 ^#§^	178.3 ± 7.5 *^§^	185.9 ± 11.2 *^#^	<0.001	181.9 ± 10.4	169.2 ± 7.7 ^§^	167.2 ± 7.9 ^§^	176.1 ± 9.9 *^#^	<0.001	171.4 ± 9.8
BMI (kg/m^2^)	21.9 ± 4.1 ^#§^	23.5 ± 2.5 ^*^	24.4 ± 4.4 ^*^	<0.001	23.7 ± 4.0	21.4 ± 2.3	22.1 ± 1.8	22.1 ± 2.3	0.087	21.9 ± 2.1
R/H (ohm/m)	267.2 ± 28.0 ^#§^	253.3 ± 32.4 *^§^	246.2 ± 32.3 *^#^	<0.001	251.6 ± 32.5	337.5 ± 42.9 ^§^	321.0 ± 46.9 ^§^	305.6 ± 37.6 *^#^	<0.001	318.1 ± 42.8
Xc/H (ohm/m)	35.5 ± 4.7 ^#§^	34.2 ± 5.5 *^§^	32.9 ± 4.8 *^#^	<0.001	33.9 ± 4.8	40.1 ± 5.5 ^§^	38.0 ± 7.4 ^§^	36.3 ± 5.3 *^#^	<0.001	38.3 ± 6.4
Z/H (ohm/m)	269.6 ± 28.1 ^#§^	255.6 ± 32.3 *^§^	248.4 ± 32.4 *^#^	<0.001	253.8 ± 32.7	338.5 ± 42.9 ^§^	326.1 ±44.8 ^§^	307.8 ± 37.7 *^#^	<0.001	320.4 ± 43.1
PA (degree)	7.6 ± 0.8	7.7 ± 0.8	7.6 ± 0.8	0.446	7.7 ± 0.8	6.8 ± 0.8	7.0 ± 0.8	6.8 ± 0.8	0.060	6.9 ± 0.8
FM (kg)	10.4 ± 3.6 ^§^	11.6 ± 4.2 ^§^	15.7 ± 5.8 *^#^	<0.001	13.7 ± 5.6	12.8 ± 6.3	12.6 ± 4.5 ^§^	14.3 ± 5.6 ^#^	0.015	13.6 ± 5.5
FM (%)	14.6 ± 3.5 ^§^	15.2 ± 3.5 ^§^	18.1 ± 3.8 *^#^	<0.001	16.7 ± 4.1	20.2 ± 8.8	19.8 ± 5.6	20.5 ± 6.2	0.719	20.2 ± 6.7
FFM (kg)	59.4 ± 5.3 ^#§^	63.2 ± 6.2 *^§^	68.9 ± 8.8 *^#^	<0.001	65.7 ± 8.5	48.4 ± 5.5 ^§^	49.9 ± 5.5 ^§^	54.6 ± 6.2 *^#^	<0.001	51.9 ± 6.4
TBW (L)	43.9 ± 4.5 ^#§^	47.3 ± 5.3 *^§^	51.8 ± 7.5 *^#^	<0.001	49.3 ± 7.3	35.2 ± 4.2 ^§^	36.6 ± 4.4 ^§^	39.8 ± 4.8 *^#^	<0.001	38.0 ± 4.9

ANOVA: analysis of variance; BMI: body mass index; R/H: resistance standardized for height; Xc/H: reactance standardized for height; Z/H: vector length standardized for height; PA: phase angle; FM: fat mass; FFM: fat-free mass; TBW: total body water. * Differences (*p* < 0.017) compared with the endurance group. ^#^ Differences compared with the velocity/power group. ^§^ Differences compared with the team-sports group. All post hoc Bonferroni test, 1-way ANOVA.
